# Hamstring Muscle Stiffness in Athletes with and without Anterior Cruciate Ligament Reconstruction History: A Retrospective Study

**DOI:** 10.3390/jcm13154370

**Published:** 2024-07-26

**Authors:** Ersagun Kepir, Furkan Demiral, Esedullah Akaras, Ahmet Emre Paksoy, Buket Sevindik Aktas, Bahar Yilmaz Cankaya, Bilgehan Oztop, Gokhan Yagiz, Julian Andrew Owen

**Affiliations:** 1 Institute for Applied Human Physiology, School of Psychology and Sport Sciences, Bangor University, Bangor LL57 2DG, UK; j.owen@bangor.ac.uk; 2 Department of Radiology, Faculty of Medicine, Ataturk University, 25240 Erzurum, Türkiye; 3 Department of Physiotherapy and Rehabilitation, Faculty of Health Sciences, Erzurum Technical University, 25050 Erzurum, Türkiye; 4 Department of Orthopaedic Surgery, Faculty of Medicine, Ataturk University, 25240 Erzurum, Türkiye; 5 Faculty of Sport Sciences, Erzurum Technical University, 25050 Erzurum, Türkiye; 6 Department of Physical Medicine and Rehabilitation, Faculty of Medicine, Ataturk University, 25240 Erzurum, Türkiye; 7 Department of Kinesiology, College of Health and Human Performance, East Carolina University, Greenville, NC 27858, USA; 8 Department of Physiotherapy and Rehabilitation, Faculty of Health Sciences, Amasya University, 05100 Amasya, Türkiye; 9 Department of Physical Therapy, Faculty of Health Sciences, Tokyo Metropolitan University, Tokyo 192-0397, Japan

**Keywords:** posterior thigh, ligament injuries, lower limb, sonoelastography, elasticity, muscle hardness, muscle tenderness

## Abstract

**Introduction:** Sports requiring sprinting, jumping, and kicking tasks frequently lead to hamstring strain injuries (HSI). One of the structural risk factors of HSI is the increased passive stiffness of the hamstrings. Anterior cruciate ligament (ACL) injury history is associated with a 70% increase in the incidence of HSI, according to a recent meta-analysis. The same report recommended that future research should concentrate on the relationships between the HSI risk factors. Hence, the present study aimed to retrospectively compare changes in the passive stiffness of the hamstrings in athletes with and without ACL reconstruction history. **Methods:** Using ultrasound-based shear-wave elastography, the mid-belly passive muscle stiffness values of the biceps femoris long head, semimembranosus, and semitendinosus muscles were assessed and compared amongst athletes with and without a history of ACL reconstruction. **Results:** There were no significant differences in the biceps femoris long head (injured leg (IL): 26.19 ± 5.28 KPa, uninjured contralateral (UL): 26.16 ± 7.41 KPa, control legs (CL): 27.64 ± 5.58 KPa; IL vs. UL: *p* = 1; IL vs. CL: *p* = 1; UL vs. CL: *p* = 1), semimembranosus (IL: 24.35 ± 5.58 KPa, UL: 24.65 ± 8.35 KPa, CL: 22.83 ± 5.67 KPa; IL vs. UL: *p* = 1; IL vs. CL: *p* = 1; UL vs. CL, *p* = 1), or semitendinosus (IL: 22.45 ± 7 KPa, UL: 25.52 ± 7 KPa, CL: 22.54 ± 4.4 KPa; IL vs. UL: *p* = 0.487; IL vs. CL: *p* = 1; UL vs. CL, *p* = 0.291) muscle stiffness values between groups. **Conclusions:** The passive mid-muscle belly stiffness values of the biceps femoris long head, semitendinosus, and semimembranosus muscles did not significantly differ between previously injured and uninjured athletes; therefore, further assessment for other muscle regions of hamstrings may be necessary. To collect more comprehensive data related to the structural changes that may occur following ACL reconstructions in athletes, a future study should examine the passive stiffness of wider muscle regions from origin to insertion.

## 1. Introduction

Hamstring strain injuries (HSIs) are widespread in sports that include running, jumping, and kicking tasks [1,2,3,4,5,6,7,8,9,10], and epidemiologic data suggest that the incidence of these injuries has been increasing over time [11,12,13,14,15]. Hamstring strain re-injuries [2,15] are also more severe than the initial injury [12,15]. Together, these findings have led to much focus on injury prevention strategies for HSIs over the last two decades [15,16,17,18].

Regarding the HSI mechanism, the most vulnerable time is defined as the late swing phase of running for the hamstrings [19,20,21]. At this moment, hamstrings generate eccentric force to control the antagonist knee extensors and decelerate the tibia [22]. During the late swing phase of running, the positions of the hip and knee joints and eccentric tensile force elongate the hamstring muscles (biceps femoris: +9.5%, semitendinosus: +8.1%, semimembranosus +7.4%) [23]. Due to this stretching, HSIs frequently occur when the eccentric actions of the hamstring muscles’ fibers cannot withstand the increased tensile force [24].

Studies have defined various HSI risk factors and classified them as modifiable and non-modifiable [15,25,26]. For example, older age [27,28,29,30,31] and previous injuries, such as calf strains [32,33,34,35,36], knee injuries [37,38], ankle ligament injuries [39], and anterior cruciate ligament injuries [32,33], are defined as non-modifiable risk factors for HSIs [26]. Conversely, modifiable risk factors include, but are not restricted to, lower hamstring strength qualities [37,40,41,42], shorter single-leg hop distance [41], higher differences between countermovement jumps and non-countermovement jumps [43], increased electromyographic (EMG) activity of the gluteus medius muscle [44], lower EMG activity of the trunk muscles [45], elevated high-speed running exposure [46,47], thoracic bending at the front-swing phase of sprinting [48], increment in anterior pelvic tilt at the backswing phase of sprinting [48], and structural risk factors (such as shorter biceps femoris long head fascicle lengths (BFlh FL) [49], and increased passive stiffness of the hamstrings) [50]. Research on muscle structure, including the increased passive muscle stiffness of the hamstrings [26], has received great attention related to sports performance [51,52,53,54,55,56,57,58,59,60,61,62,63,64,65,66,67,68,69,70,71,72,73,74] and musculoskeletal injuries [49,50,75,76,77,78,79,80,81,82,83,84,85,86]. Besides this information, a recent meta-analytic study identified that an anterior cruciate ligament (ACL) injury history causes a 70% increment in the risk of HSI [26]. The same meta-analytic study [26] suggested that future studies should focus on interactions between risk factors for HSIs.

Previously, studies examining passive muscle stiffness were performed using old techniques, such as free oscillation [50], which measures whole knee flexors’ muscle and tendon units together and does not allow for the measurement of the stiffness of specific muscles. With advancements in technology, examining a specific muscle or tendon’s passive stiffness has become possible by using ultrasound shear-wave elastography, which is both valid and reliable [86,87,88,89,90,91]. In this way, gathering specific information on each hamstring muscle’s stiffness in athletes who have returned to play from HSIs can provide new insights into the hamstring injury rehabilitation process.

Previous retrospective studies examining muscle stiffness values after ACL reconstructions have found conflicting results in the literature [92,93]. Kuszewski et al. [93] reported a considerable increment in the stiffness of the hamstrings after ACL reconstructions by using passive knee extension in the supine position [94,95]. Contrarily, He et al. [92] found a decrease in the passive stiffness of the semimembranosus and semitendinosus muscles, but no change in the stiffness of the biceps femoris. However, both the studies were conducted on non-athletic groups [92,93]. Therefore, this study aimed to retrospectively examine the alterations in the hamstring muscles’ passive stiffness in athletes with and without ACL injury history. This study hypothesized that there would be an increment in the passive stiffness values of the hamstring muscles, especially in the semitendinosus, due to possible scar tissue after surgery.

## 2. Methodology

### 2.1. Research Design and Participants

A retrospective, comparative case–control study design was used in this study. Thirteen athletes with previous ACL injuries (time since the surgery, 13.8 ± 7.1 months) and ACL reconstruction surgery history (*n* = 13), as well as twenty previously uninjured athletes (*n* = 20), participated in this study. The previously injured legs of the athletes with ACL injury history constituted the injured legs group (*n* = 13). The uninjured legs of the athletes with ACL injury history constituted the internal control group (*n* = 13). Both legs of the injury-free athletes were used as the external control group (*n* = 40). Ethical approval was obtained from the Ataturk University Sport Sciences Ethics Committee on 23 November 2022 (Approval No: E-70400699-000-2200385479). According to the Declaration of Helsinki [96], participants received informed consent forms and read and signed them before their participation in the study. Data collection was completed between 23 November 2022 and 12 August 2023.

Athletes who fully returned to sports after an ACL reconstruction were included in the previously injured group. The inclusion criteria for the injured group were (a) aged between 18 and 35 years old, (b) having returned to pre-level sports competition after an ACL reconstruction surgery, and (c) semitendinosus graft being used in the reconstruction of the ACL (this criterion was for increasing the homogeneity of the surgical technique employed in the participants). The control group included participants between 18 and 35 years old without a known lower extremity injury history who participated in sports like the injured group. The injured group consisted of ten soccer players, two basketball players, and one skier. The non-injured group consisted of ten basketball players, four skiers, two soccer players, two biathletes, one tennis player, and one handball player. The injured group had received traditional physiotherapy procedures for ACL rehabilitation, while the others kept their daily routines. However, due to the nature of the retrospective research design, the details of the rehabilitation programs could not be recorded.

### 2.2. Sample Size

The present study calculated the required sample size using the G*Power software (version 3.9.7.1) for F tests/ANOVA: fixed effects. The effect size (Cohen’s *d* = 1.8) was adapted from a similar retrospective study [93] that mentioned higher hamstring muscle stiffness in participants with ACL injury history than in participants without ACL injury history. However, this study employed a smaller effect size of 0.8 to ensure greater statistical power. The other parameters used during the sample size calculations were a 0.05 alpha level, 80% statistical power, and two groups. The required total sample size was calculated as 16 (8 for the previously injured group and 8 for the uninjured group). However, this study recruited more participants (13 for the previously injured group and 20 for the uninjured group) to increase its statistical power.

### 2.3. Measurement Procedures

Before the assessment of the participants’ ages, physical characteristics (height (cm), height of the legs (cm), and body mass (kg)) and time since returning to sports after the ACL injury were recorded. The preferred kicking leg was accepted as the dominant leg.

### 2.4. Hamstring Muscles’ Passive Stiffness Measurements

**Figure 1:**
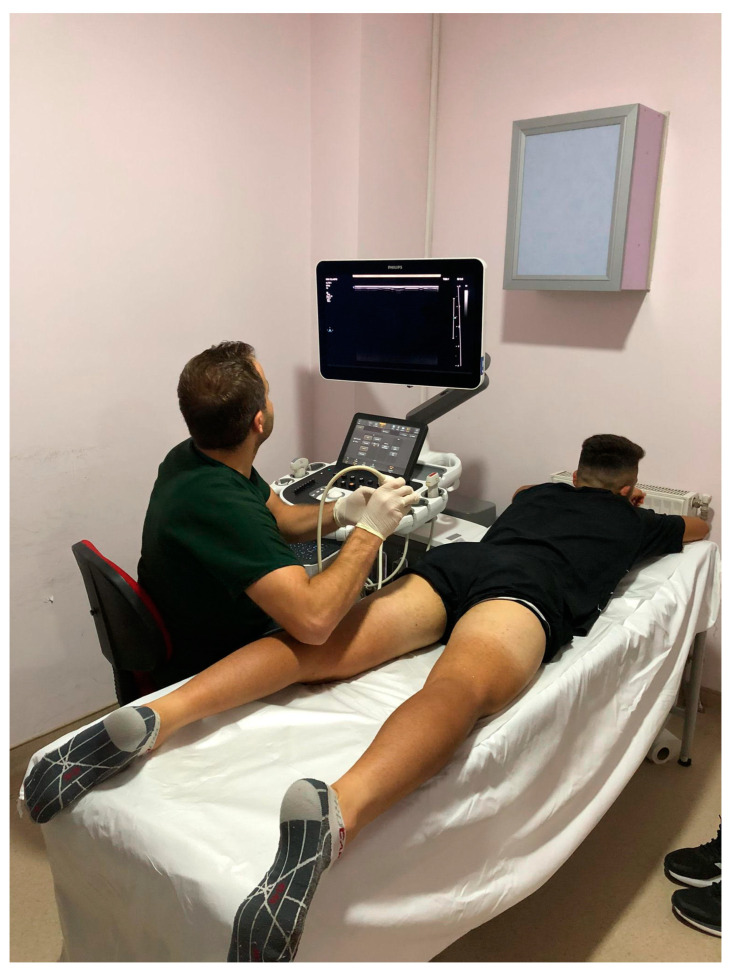
Participants’ position on a medical bed during the shear-wave elastography measurements.

**Figure 2:**
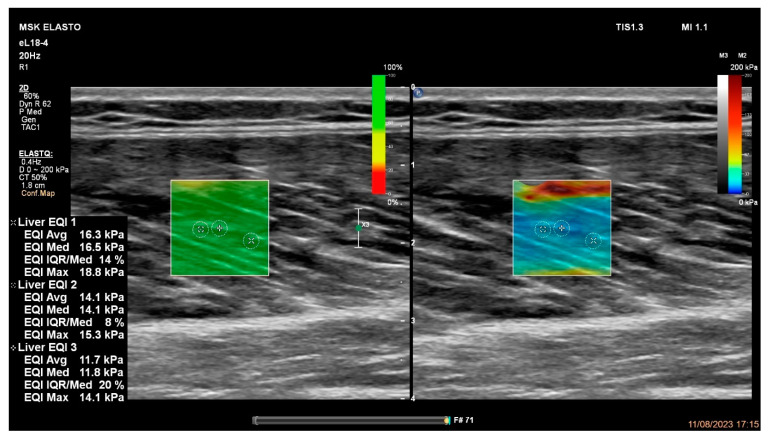
An example of the semimembranosus muscle stiffness measurement using ultrasound-based shear-wave elastography.

Participants laid down on a medical bed in a prone position with zero degrees of hip and knee flexion without any voluntary muscle contractions (Figure 1). The ultrasound machine’s (Phillips EPIQ Elite, Phillips Ultrasound Systems, Amsterdam, The Netherlands) transducer (PureWave, eL18-4, Phillips Ultrasound Systems, Amsterdam, The Netherlands) was placed on the muscle belly (50% of the distance between the proximal and distal musculotendinous junctions) [91] parallel to the orientations of each BFlh, semimembranosus, and semitendinosus muscle. The probe was arranged parallel to the muscle fascicle orientations for more accurate results [87,97]. A slight and equal transducer pressure was applied during the measurements to avoid misinterpretations of the results [98]. The region of interest (ROI) was selected in the center of the BFlh, semimembranosus, and semitendinosus muscles [91]; three random elastograms were taken from the ROI; and the mean value was accepted as the muscle stiffness of each muscle. As a visual example, a biceps femoris long-head muscle stiffness measurement is illustrated in Figure 2. The measurements were repeated for both muscles of both legs in all participants by an experienced radiology clinician (the second author: FD). All the assessments were performed in the same room conditions (24 degrees Celsius) on the same medical bed.

### 2.5. Statistical Analyses

The one-way analysis of variance (ANOVA) was used to compare passive stiffness scores between the injured leg (*n* = 13), the uninjured contra-lateral leg (n = 13), and both legs together in the control group (*n* = 40). The homogeneity of variance between the groups was compared using Levene Statistics. The alpha value was set at 0.05 for the indicator of statistical significance. All the statistical analyses were performed via IBM SPSS Statistics, version 29.0.

## 3. Results

Thirteen athletes with an ACL injury history (age, 24 ± 4.2 years; body mass, 79.2 ± 9.5 kg; height, 176.5 ± 5.8 cm; time since the surgery, 13.8 ± 7.1 months) and twenty athletes without an ACL injury history (age, 19.7 ± 2.1 years; body mass, 75.6 ± 11.8 kg; height, 182.2 ± 9 cm) participated to this study. Eight ACL injuries occurred in the non-dominant leg, while five ACL injuries occurred in the dominant leg. Based on the one-way ANOVA, there were no significant differences in any passive stiffness scores of the BFlh, semimembranosus, or semitendinosus muscles between the injured legs and the uninjured contralateral legs, or between the injured legs and the control group’s legs (Table 1).

## 4. Discussion

This study aimed to retrospectively examine the alterations in the hamstring muscles’ passive stiffness in athletes with and without ACL injury history. The main findings of our study showed that there were no significant alterations in the passive stiffness of the BFlh, semimembranosus, or semitendinosus mid-muscle bellies between injured and non-injured limbs using ultrasound-based shear-wave elastography.

Changes in tissue (i.e., mechanical properties) may affect the passive tension and stiffness of muscles, causing them to exhibit higher values [86,99]. After lower-extremity injuries, higher stiffness has been observed in previous studies [86,93,100]. Considering possible scars in the surgery area, which can increase muscle stiffness [100,101,102], this study hypothesized a possible increment in muscle stiffness. However, the findings of previous retrospective studies examining muscle stiffness values after ACL reconstruction are conflicting in the literature [92,93]. Kuszewski et al. [93] reported a considerable increase in the stiffness of the hamstrings after ACL reconstruction by using passive knee extension in the supine position [94,95]. Conversely, He et al. [92] suggested decreases in passive stiffness of the semimembranosus and semitendinosus muscles, but no change in the stiffness of the biceps femoris using 11 mm central elastograms via shear-wave elastography. These contradictory findings might be caused by differences between these two techniques [92,93]. The technique of Kuszewski et al. [93] measured whole knee flexor muscles and tendons together, which indicated an increase in the whole knee flexor properties together. However, He et al. [92] measured the stiffness of the hamstrings within an 11 mm circle located at the muscle bellies of the hamstrings. Therefore, increased stiffness may be detected at the distal regions of the hamstrings, which may be logical when considering that the semitendinosus tendon grafts are usually taken from the distal tendons for ACL surgeries [103,104]. This may suggest that increased stiffness may be detected around the distal tendons in consideration of previous observations, suggesting increased tendon values after tendon injuries [100]. Muscle stiffness values can be affected by measurement depths and the size of range of interest values [105]. Regarding our findings of no difference in passive hamstring stiffness after ACL injury, our measurements consisted of three smaller (3 mm) points only in the blue-colored area (Figure 2). However, the shear-wave elastography used in this study included a color-mapping mechanism to show the stiffness differences of the structures, with the blue color representing the lowest stiffness values in the tissue. The assessor of this study took all the measurements as small points from the blue-colored areas of the muscle bellies, as shown in Figure 2, which is a limitation of this study in terms of detecting the stiffness of whole muscles. Overall, this study’s strength is that it was conducted on athletes, as compared to previous relevant studies. Despite this, this study suggests that rehabilitation specialists and coaches may consider employing elastography to monitor the healing process of soft tissues after sports injuries.

In addition to the depth and size of the range of interest [105], hamstring muscle stiffness results may be influenced by muscle size [91], sex [106], sports profession [91,107], pelvic tilt type [108], hip and knee positions [109], injury and scar status [100,101,102], the assessor of the shear-wave elastography [110], age [111,112], and genetic factors [111,113]. Moreover, there may be differences in the measurement algorithms of the ultrasound-based shear wave elastography; for example, the values of the BFlh passive muscle belly stiffness at rest have been stated to be 4.5 kPa [114], 9.91 kPa (this study), 10.54–15.72 [115], 10.8 kPa [116], 11.3–11.7 kPa [117], 11.57 kPa, [118], 14.43–16.27 kPa [119], 15.74–19.01 kPa [90], 16.47–19.87 kPa [120], and 16.9–24.7 kPa [91] by different studies in the literature. Hence, a wider study comparing the measurement values of the commercially available devices is necessary in order to clarify different results in the literature. Among the factors mentioned above, the assessor of the shear-wave elastography [110] may be necessary to mention for this study. Despite our measurements being performed by a radiologist, this study did not include a reliability study, which can be considered one of the study’s major limitations, requiring cautious interpretation of our outcomes.

Among the other limitations is the fact that retrospective studies do not permit conclusions about the cause and effect of the outcome measures. An additional limitation of this research design is that it did not gather detailed information about the healing process after injury, nor about treatment and rehabilitation after ACL injuries. Additionally, the assessor was not blinded to the participants’ groups, which is another limitation of this study. As mentioned above, the lack of reliable analyses (only muscle belly stiffness was analyzed) and the lack of assessor-blinded status are other limitations of this study, which add accountable confounding factors to the results. Moreover, the pressure which was applied by the assessor is unknown and was not measured before the study, which is another important limitation of this study. Furthermore, conducting this study with only male participants is another limitation which decreases its generalizability, considering the higher rate of ACL injuries in females [121]. A future study on females should focus on the same topic as this study. Additionally, a randomized controlled study should observe the effectiveness of rehabilitation programs in improving muscle stiffness by applying repeated measures.

## 5. Conclusions

This study aimed to retrospectively compare the alterations in the hamstring muscles’ passive stiffness in athletes with and without a history of ACL injury. However, no significant differences were observed between previously injured and uninjured athletes’ passive mid-muscle belly stiffness values of the biceps femoris long head, semitendinosus, or semimembranosus muscles. A future study should investigate the passive stiffness of larger areas in the muscles from origin to insertion in order to gather broader data with which to illuminate structural alterations after ACL reconstructions in athletes. Rehabilitation specialists should consider employing elastography to monitor the healing process of soft tissues after sports injuries.

## Figures and Tables

**Table 1:** Differences in the passive stiffness of BFlh, semitendinosus, and semimembranosus muscle between injured thighs, uninjured contralateral thighs, and control thighs (kPa).

	Injured Thighs (*n* = 13) (mean ± SD)	Uninjured Contralateral Thighs (*n* = 13) (Mean ± SD)	Control Thighs (*n* = 40) (Mean ± SD)	*p*-Values -Injured vs. Contralateral -Injured vs. Control -Contralateral vs. Control
**BFlh**	26.19 ± 5.28	26.16 ± 7.41	27.64 ± 5.58	Injured vs. contralateral: *p* = 1 Injured vs. control: *p* = 1 Contralateral vs. control: *p* = 1
**Semimembranosus**	24.35 ± 5.58	24.65 ± 8.35	22.83 ± 5.67	Injured vs. contralateral: *p* = 1 Injured vs. control: *p* = 1 Contralateral vs. control: *p* = 1
**Semitendinosus**	22.45 ± 7	25.52 ± 7	22.54 ± 4.4	Injured vs. contralateral: *p* = 0.487 Injured vs. control: *p* = 1 Contralateral vs. control: *p* = 0.291

Abbreviations. BFlh, biceps femoris long head; SD, standard deviation.

## Data Availability

Data is contained within the article or Supplementary Material. The data presented in this study are available in Manuscript-Supplementary.xlsx.

## Supplementary Materials

The following supporting information can be downloaded at: https://www.mdpi.com/article/10.3390/jcm13154370/s1.
